# Hypoxemic reperfusion of ischemic states: an alternative approach for the attenuation of oxidative stress mediated reperfusion injury

**DOI:** 10.1186/s12929-016-0220-0

**Published:** 2016-01-19

**Authors:** Marios–Konstantinos Tasoulis, Emmanuel E. Douzinas

**Affiliations:** 2nd Department of Surgery, National and Kapodistrian University of Athens, Medical School, Aretaieion University Hospital, 76 Vas. Sofias Ave, 11528 Athens, Greece; 3rd Department of Critical Care Medicine, National and Kapodistrian University of Athens, Medical School, Evgenideio Hospital, 20 Papadiamantopoulou St., 11528 Athens, Greece

**Keywords:** Reperfusion injury, Hypoxemic reperfusion, Oxidative stress, Reactive oxygen species

## Abstract

Ischemia and reperfusion (I/R) – induced injury has been described as one of the main factors that contribute to the observed morbidity and mortality in a variety of clinical entities, including myocardial infarction, ischemic stroke, cardiac arrest and trauma. An imbalance between oxygen demand and supply, within the organ beds during ischemia, results in profound tissue hypoxia. The subsequent abrupt oxygen re-entry upon reperfusion, may lead to a burst of oxidative aggression through production of reactive oxygen species by the primed cells. The predominant role of oxidative stress in the pathophysiology of I/R mediated injury, has been well established. A number of strategies that target the attenuation of the oxidative burst have been tested both in the experimental and the clinical setting. Despite these advances, I/R injury continues to be a major problem in everyday medical practice. The aim of this paper is to review the existing literature regarding an alternative approach, termed hypoxemic reperfusion, that has exhibited promising results in the attenuation of I/R injury, both in the experimental and the clinical setting. Further research to clarify its underlying mechanisms and to assess its efficacy in the clinical setting is warranted.

## Background

Tissue hypoxia due to ischemia is the common denominator in a variety of clinical emergencies of either regional distribution such as myocardial infarction and mesenteric embolism, or of systemic involvement such as cardiac arrest and hemorrhagic shock, both of which represent the equivalent of whole body ischemia.

The aforementioned conditions are sometimes fatal because of the injury that lurks to appear, the so called ischemia – reperfusion (I/R) injury. This represents somehow, the cost of optimal reperfusion or effective resuscitation from longstanding insults of ischemia. Reperfusion injury does not occur during the preceding ischemic period; rather, this injury refers to a causal event associated with reperfusion. This event may result in a number of detrimental effects including multiple organ failure (MOF) and death. Over the last several decades, a variety of treatment modalities have been evaluated to attenuate I/R injury. Hypoxemic reperfusion, in particular, may be particularly promising. The aim of this review is to summarize the existing literature regarding the underlying mechanisms and potential applications of hypoxemic reperfusion in a variety of clinical scenarios of regional and systemic I/R injury.

## Review

### Strategies of reperfusion injury prevention

Reactive oxygen species have been found to play a key role in the pathophysiology of I/R injury [1]. Oxidative stress generated during reperfusion, may mediate injury to the insulted tissues. This phenomenon is part of the term «oxygen paradox», in which reoxygenation of an ischemic tissue produces a degree of injury that greatly exceeds the injury induced by ischemia alone [2]. However, oxidative stress, contributes to I/R injury – induced damage in a consecutive two – phase pattern. In addition to its direct cytotoxic effects, the burst of free radicals, generated by oxidative stress, also induces the formation of inflammatory mediators [3] through redox-mediated signalling pathways, leading to post ischemia – reperfusion inflammatory injury [2, 3]. These oxidative and inflammatory responses have been implicated in the development of MOF, a detrimental manifestation often following I/R.

The above knowledge has led medical research to focus on the development of potential strategies aimed at eliminating the effects of reactive oxygen species (ROS) and of the systemic inflammatory response during reperfusion. Suggested methods include:

- use of antioxidants in order to minimize the oxidative stress [4–7]
- scavengers for the removal of metabolic waste
- preconditioning techniques (ischemic, hypoxic, pharmacologic and remote ischemic preconditioning) to prepare cells to better respond to the forthcoming stress [8–17]

Despite the proven beneficial effects, all the above strategies share one common disadvantage: they lack effectiveness when they are applied after or during reperfusion/resuscitation. This limits their usefulness in the clinical setting. Antioxidants should be administered ideally before ischemia and reperfusion in order to achieve their maximum effect. In fact, most available evidence regarding their favorable effects derives from studies in which antioxidants were used as pre-treatment [5, 7]. Moreover, their use, even in combination with scavengers, does not completely abolish the ensuing injury. The same applies for the use of preconditioning techniques. The rationale of these techniques is to pre-medicate the patient, which may not be feasible in all clinical scenarios. Therefore, the application of these strategies in the clinical setting may be limited [14–16, 18, 19]. A recent meta-analysis questioned the efficacy of ischemic preconditioning in the setting of liver surgery [20]. Similarly, remote ischemic preconditioning, a technique that held great promise for its demonstrated favorable effects, did not exert the expected outcomes when tested in clinical trials [21–23].

Despite the controversial results in the clinical setting, the existing literature regarding these strategies provides additional evidence, confirming the oxidative nature of the injury following reperfusion. The pivotal role of oxidative stress mediated reperfusion injury has been well established. Using a rabbit experimental model, it was shown that resuscitation from hemorrhagic shock resulted in acute lung injury with enhanced oxidative and inflammatory pulmonary responses. However, the degree of injury correlated only with the extent of oxidative aggression [24].

### Hypoxemic reperfusion

Given that the abundance of oxygen supply, initially during reperfusion, produces a burst of ROS generation, the important question was whether this phenomenon could be attenuated by manipulating the oxygen content in the initial blood perfusate in order to meet with the adapted -at low cellular energy level- needs during ischemia.

Over the last years, a growing body of literature examines the effect of ischemic post-conditioning, which seems to be a promising approach [25–30]. This method is based on the concept that gradual reperfusion of a previously ischemic tissue, interrupted with short-lived episodes of ischemia, might yield favorable results. A closely related technique, termed “remote ischemic post-conditioning”, involves initiation of transient episodes of ischemia in a remote tissue or organ at the time of reperfusion [31–34]. A third, relatively new approach, similar to these is hypoxic post-conditioning, characterized by reperfusion under normoxia alternated with periods of hypoxia [35–38].

This is where hypoxemic reperfusion appears. It is about gradually increasing the FiO_2_ of the reperfusate from a lower level in order to maintain P_a_O_2_ levels of 30 – 35 mmHg, initially during reperfusion, to gradually achieve P_a_O_2_ levels of 95 – 105 mmHg at the end of the resuscitation period. Historically, the accepted dogma was to give as much oxygen as possible to treat ischemic states. However, to deliver oxygen in plenty, particularly early in reperfusion, may only lead to higher quantities of ROS. Therefore, the imperative is to supply sufficient oxygen to meet tissue oxygen demand to maintain vital functions while minimizing reperfusion injury related to an abundance of ROS. Experimental findings indicate a significant correlation between P_a_O_2_ and the phosphocreatine/inorganic phosphate ratio or intracellular pH. However, for P_a_O_2_ ranging from 130 to 33 mmHg, metabolite changes were not significant. Both the ratio as well as the intracellular pH decreased significantly when P_a_O_2_ was lowered below 33 and 28 mmHg respectively [39]. Moreover, the seemingly paradoxical idea of hypoxemic reperfusion is very similar to the aforementioned strategies of post-conditioning in terms of physiology. What is common in these two methods is the lower delivery of oxygen early during reperfusion so as not to provide it in abundance to form ROS. How is this possible? Delivery of oxygen is calculated by the following formula: DO_2_ = CO × C_a_O_2_ (C_a_O_2_ = 13.4 × [Hb] × S_a_O_2_ + 0.03 P_a_O_2_). During post-conditioning the altered parameter is the cardiac output (CO) through the gradual reperfusion whereas during hypoxemic reperfusion the altered parameter is S_a_O_2_ and P_a_O_2_ through the gradual increase of FiO_2_.

**Fig. 1:**
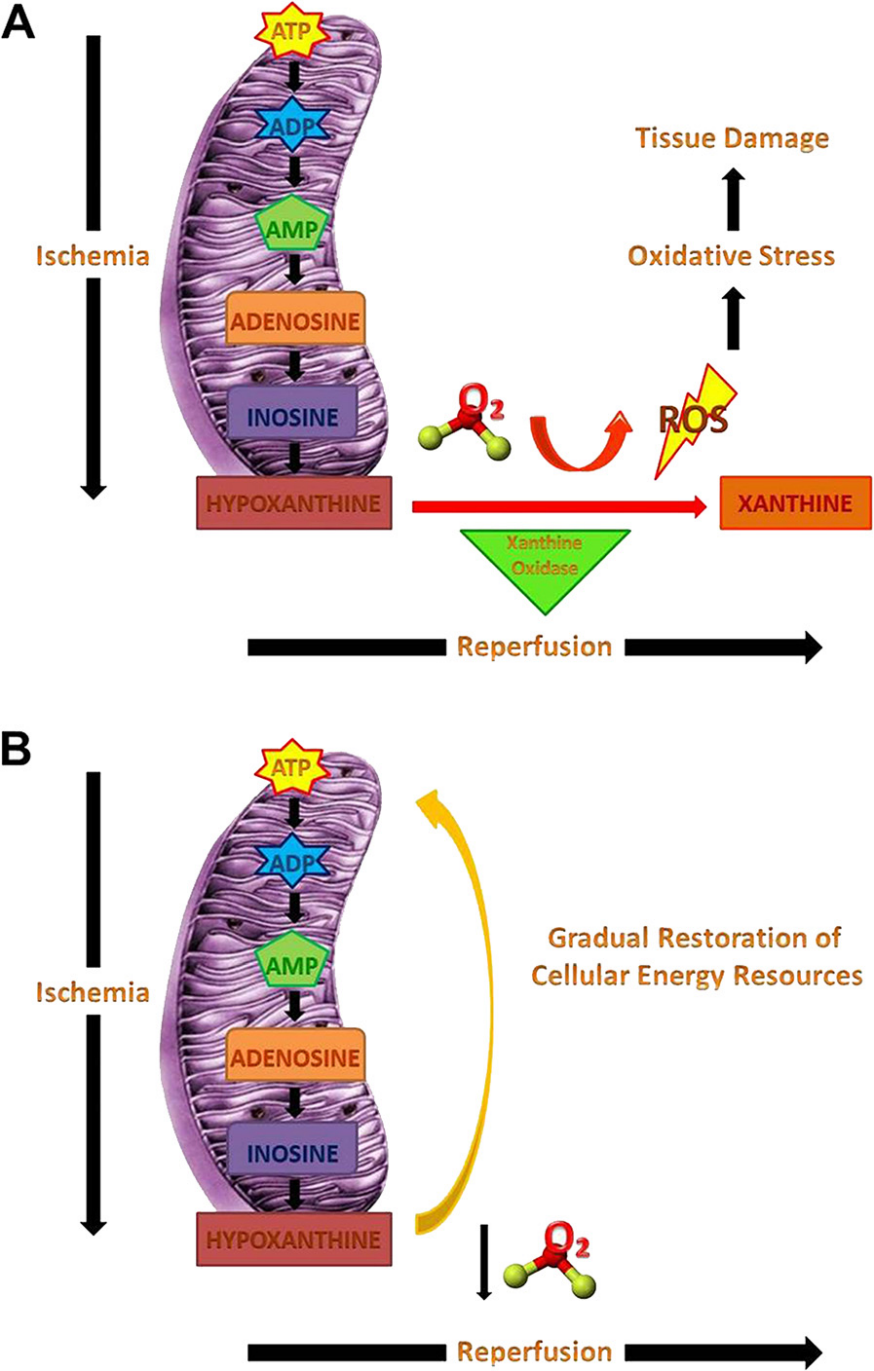
Representation of biochemical events that lead to the production of reactive oxygen species (ROS) and subsequent tissue damage during ischemia and reperfusion (Panel **a**). Panel **b** represents the hypothesis of restoration of cellular energy resources achieved by reperfusion of the previously ischemic tissues under lower P_a_O_2_ (hypoxemic reperfusion) with gradual return to normoxemia

The basic underlying concept of hypoxemic reperfusion is that when reperfusion of a previously ischemic tissue takes place under hypoxemia, the cells will not be supplied with an abundance of oxygen, which will be available for ROS production (Fig. 1a). Alternatively, it is hypothesized that the gradual reintroduction of oxygen in increasing concentration during reperfusion, corresponding to the increasing tissue requirements, may be used by the mitochondria for the generation of ATP and the restoration of the cell’s energy resources (Fig. 1b). Therefore, hypoxemic reperfusion represents a strategy that can be applied after the occurrence of the ischemic insult, at the beginning of reperfusion. This could be a significant advantage when compared to other reperfusion strategies, including the use of antioxidants and preconditioning, since these techniques need to be implemented before reperfusion to show their beneficial effects [5, 7, 14–16]. Another potential advantage of hypoxemic reperfusion compared to the use of antioxidants, is that it aims to prevent ROS production rather than eliminate their deleterious effects. Moreover, hypoxemic reperfusion may be advantageous compared to post-conditioning strategies since blood flow is restored offering better replenishment from metabolic wastes. However, apart from the theory, the method should be examined in vivo. Indeed the existing evidence, summarized in Table 1, exhibit remarkably favorable results in various applications both in the experimental and the clinical setting.

**Table 1:** Summary of hypoxemic reperfusion studies

Study	Type of study	Model of ischemia	Reperfusion protocol	Outcome
Perry et al. [40]	Experimental	Celiac artery ischemia through adjustable screw clamp	P_a_ O_2_ = 34 mmHg for 1 h before return to normal perfusion	↓gastric mucosal bleeding
Douzinas et al. [41]	Experimental	SMA clamping	P_a_ O_2_ = 30–35 mmHg with gradual return to normoxemia over a 2 h period	↓intestinal mucosa and lung injury
				↓inflammatory response
Douzinas et al. [42]	Experimental	SMA clamping	P_a_ O_2_ = 30–35 mmHg with gradual return to normoxemia over a 2 h period	↑hemodynamic profile
				↓oxidative response
				↓myocardial injury
Burda et al. [43]	Experimental	Clamping of left subclavian artery and brachiocephalic trunk	P_a_ O_2_ = 37.5 mmHg with gradual return to normoxemia over a 15–30 minute period	↑cerebral protein synthesis
Douzinas et al. [44]	Experimental	Global cerebral ischemic insult through decrease of MAP, bilateral clamping of carotid arteries and cessation of respiration	FiO_2_ = 0.12 with gradual increase to achieve P_a_O_2_ = 100 mmHg over a 1 h period	↑neurological outcome
				↓oxidative response
Douzinas et al. [45]	Experimental	Global cerebral ischemic insult through decrease of MAP, bilateral clamping of carotid arteries and cessation of respiration	P_a_ O_2_ = 30–35 mmHg with gradual increase to achieve P_a_O_2_ = 100 mmHg over a 1 h period	↓cerebral injury
Hickey et al. [46]	Experimental	Deep hypothermic circulatory arrest	P_a_ O_2_ = 40–50 mmHg throughout the reperfusion period	↑cerebral injury
Abdel-Rahman et al. [47]	Experimental	Aortic clamping and cardioplegic arrest	P_a_ O_2_ = 40–50 mmHg gradually increased towards normoxemia over a 10 minute period	↑hemodynamic profile
				↓myocardial injury
				↓oxidative response
Abdel-Rahman et al. [48]	Clinical	CPB for CABG	P_a_ O_2_ = 50 mmHg with return to normoxemia over a 5 minute period	↓oxidative response
Fercakova et al. [49]	Experimental	Infrarenal aortic occlusion	Graded postischemic reoxygenation	↑neuroprotection
Daxnerova et al. [50]	Experimental	Infrarenal aortic occlusion	Graded postischemic reoxygenation	↑neuroprotection
Marsala et al. [51]	Experimental	Infrarenal aortic occlusion	Graded postischemic reoxygenation	↓neuropathological damage
Orendacova et al. [52]	Experimental	Infrarenal aortic occlusion	P_a_ O_2_ = 48 mmHg with gradual return to normoxemia over a 15 minute period	↑neuroprotection
Lukacova et al. [53]	Experimental	Infrarenal aortic occlusion	P_a_ O_2_ = 48 ± 12 mmHg with gradual return to normoxemia over a 30 minute period	↑neuroprotection
Lehmann et al. [54]	Experimental	Supraceliac aortic clamp	P_a_ O_2_ = 25–35 mmHg for 30 minutes with gradual return to normoxemia over a 90 minute period	↓hemodynamic profile
Douzinas et al. [55]	Experimental	Hemorrhagic shock - exsanguination	FiO_2_ = 0.12 with gradual increase to FiO_2_ = 0.21 over a 40 minute period	↑hemodynamic profile
				↓oxidative response
				↓inflammatory response
Douzinas et al. [3]	Experimental	Hemorrhagic shock - exsanguination	FiO_2_ = 0.08–0.10 with gradual increase to FiO_2_ = 0.21 over a 60 minute period	↑hemodynamic profile
				↓oxidative response
				↓inflammatory response
Douzinas et al. [56]	Experimental	Hemorrhagic shock - exsanguination	FiO_2_ = 0.08–0.10 with gradual increase to FiO_2_ = 0.21 over a 60 minute period	↓oxidative response
				↓inflammatory response
Douzinas et al. [57]	Experimental	Hemorrhagic shock - exsanguination	FiO_2_ = 0.08-0.10 with gradual increase to FiO_2_ = 0.21 over a 60 minute period	↑vascular homeostasis
Douzinas et al. [58]	Experimental	Hemorrhagic shock - exsanguination	FiO_2_ = 0.08-0.10 with gradual increase to FiO_2_ = 0.21 over a 60 minute period	↓oxidative response
				↓lung injury
Douzinas et al. [59]	Experimental	Hemorrhagic shock - exsanguination	FiO_2_ = 0.08-0.10 with gradual increase to FiO_2_ = 0.21 over a 60 minute period	↓oxidative response
				↓inflammatory response
				↓lung injury
Douzinas et al. [60]	Experimental	Hemorrhagic shock - exsanguination	FiO_2_ = 0.08-0.10 with gradual increase to FiO_2_ = 0.21 over a 60 minute period	↓oxidative response
				↓inflammatory response
				↓liver injury
Luo et al. [61]	Experimental	Hemorrhagic shock - exsanguination	FiO_2_ = 0.11 with gradual increase to FiO_2_ = 0.21 over a 60 minute period	↓hemodynamic profile
				─oxidative response
				─inflammatory response

Table summarizes the data of the available studies of hypoxemic reperfusion presenting the setting, the model of ischemia – reperfusion injury studied, the reperfusion protocol and the main outcomes

*P*_*a*_ O_*2*_ partial arterial oxygen pressure, *SMA* superior mesenteric artery, *FiO*_*2*_ fraction of inspired oxygen, *MAP* mean arterial pressure, *CPB* cardio-pulmonary bypass, *CABG* coronary artery bypass grafting

#### Gastrointestinal tract reperfusion injury

Ischemia – reperfusion induced injury of the gastrointestinal tract can ensue from a variety of clinical conditions such as mesentery artery embolism. In 1988, Perry et al. showed, for the first time, that gradual reintroduction of oxygen reduced reperfusion injury as evidenced by decreased gastric mucosal bleeding after ischemia in a cat experimental model [40].

A subsequent set of experiments, further demonstrated the favorable effect of hypoxemic reperfusion in the setting of I/R injury of the gastrointestinal tract. Specifically, using a porcine model of intestinal ischemia, through clamping of the superior mesenteric artery [41, 42], it was shown that hypoxemic reperfusion resulted in decreased gut barrier dysfunction as evidenced by the lower incidence of positive Limulus test [41]. Both the oxidative and the inflammatory responses were also attenuated as demonstrated by the decrease in intestinal mucosa malondialdehyde (MDA) [42] and portal blood interleukin (IL) -1b levels [41]. The application of this method resulted in a decrease of the observed histopathologic injury not only of the intestine [41] but also of remote organs such as the heart [42] and lung [41]. This finding implies a systemic favorable effect and highlights the potential role of hypoxemic reperfusion in the prevention of MOF through attenuation of oxidative and inflammatory responses. Furthermore, animals that underwent hypoxemic reperfusion had a superior hemodynamic profile as evidenced by mean arterial pressure preservation, lower need for inotropic support, and a trend towards S_ṽ_O_2_ restoration [42].

#### Brain reperfusion injury

The brain is thought to be the most vulnerable organ in hypoxic challenges. Compromise of cerebral perfusion and thus oxygen delivery may follow cardiac arrest, traumatic brain injury, ischemic stroke etc. In these cases, the restoration of blood flow may lead to reperfusion injury. The efficacy of gradual reintroduction of oxygen in the previously ischemic brain has been tested in a dog experimental model by Burda et al [43]. They used a global brain ischemia model produced by cross-clamping of the left subclavian artery and the brachiocephalic trunk at the point of their emergence from the aorta and showed that the animals that underwent reperfusion under hypoxemia (P_a_O_2_ = 37.5 mmHg) with gradual return to normoxemia (P_a_O_2_ = 82 mmHg) exhibited increased cerebral protein synthesis. The favorable effect of hypoxemic reperfusion has also been tested in a porcine experimental model of global cerebral ischemia [44, 45], where hypoxemic reperfusion was found to result in improved neurological outcome as evidenced by the superior Overall Performance Category (OPC) score [44], decreased brain histopathologic damage [45] and reduced lipid peroxidation [44].

There has, however, been one study in which hypoxemic reperfusion was found to exacerbate neurological injury [46]. In a porcine model designed to resemble the clinical scenario of infants undergoing deep hypothermic circulatory arrest with diminished cerebral blood flow as employed during surgical intervention for complex congenital heart lesions, investigators applied maintained hypoxemic arterial oxygen tensions (P_a_O_2_, 40 - 50 mmHg) throughout the reperfusion period without return to normoxemia [46]. While this was found to lead to worse outcomes, this method cannot be compared with other studies in which the reperfusion took place under hypoxemic conditions with gradual return to normoxemia.

#### Myocardial reperfusion injury

Myocardial reperfusion injury represents a devastating entity encountered in the clinical setting as a result of various conditions including percutaneous coronary intervention after acute myocardial infarction and cardiac surgery. The promising results of hypoxemic reperfusion have led to testing of this strategy in the setting of myocardial reperfusion injury, both in experimental and clinical studies.

Abdel – Rahman et al. used a porcine experimental model of cardiopulmonary bypass with aortic clamping and cardioplegic arrest to test the efficacy of the so-called gradual reoxygenation in the attenuation of myocardial reperfusion injury [47]. The results of their study indicate that applying hypoxemia at the onset of the reperfusion period with gradual return to normoxemia resulted in significantly less impairment of myocardial function, decreased myocardial injury and reduced oxidative damage.

The same group designed and conducted a prospective study, using nineteen consecutive patients who underwent cardiac surgery with cardiopulmonary bypass for scheduled coronary artery bypass grafting as the study’s population [48]. Graded reoxygenation at the beginning of the reperfusion period led to a decrease in myocardial oxidative injury as signified by the lower MDA blood levels.

#### Generalized ischemia - reperfusion injury

Graded post-ischemic reoxygenation has also been the topic of investigation by Maršala and his group in the setting of aortic clamping induced ischemia. Using a rabbit experimental model, they found that gradual reoxygenation during reperfusion after infrarenal aortic occlusion, resulted in the preservation of the cytoplasmic and nuclear structures of the lumbosacral dorsal root ganglia neurons [49, 50]. In a similar model of aortic clamping-induced spinal cord ischemia, they showed that the application of gradual reoxygenation reduced neuropathological damage [51], decreased blood – brain barrier permeability [52] and attenuated neuronal argyrophilia and reperfusion injury – induced alterations in neuronal organelles [53].

However, one study by Lehmann et al. using a porcine model of complete lower torso ischemia, found that hypoxemic reperfusion resulted in a deteriorated hemodynamic profile, increased lactic acidosis and higher inotropic agents requirements compared to normoxemic reperfusion [54]. A possible explanation for these findings which are discrepant from that of other studies is that in this study, there was a long aortic cross-clamp period, perhaps resulting in greater injury severity and ischemic insult.

#### Hemorrhagic shock – resuscitation

Hemorrhagic shock and resuscitation represents a model of whole body I/R injury. In a pilot study, using a rat model of controlled hemorrhagic shock, hypoxemic resuscitation resulted in superior hemodynamic stabilization and less oxidative and inflammatory responses as evidenced by the decreased MDA and tumor necrosis factor alpha (TNF-a) serum levels [55]. Similar results were shown in a rabbit experimental model. Application of hypoxemic resuscitation was associated with more efficient blood pressure restoration as well as attenuation of the oxidative insult exerted by normoxemic resuscitation [3]. This was shown by the decreased production of ROS as assessed by flow cytometry, reduced MDA and higher ratio of reduced to total glutathione levels. Moreover, hypoxemic resuscitation resulted in attenuation of the inflammatory response as evidenced by the significantly lower serum levels of TNF-a, IL-1b and IL-6. These effects could be attributed to the lower stimulation of p38 mitogen activated protein kinase (MAPK) – mediated production of inflammatory cytokines by monocytes [56]. Additionally, using this combined in vivo and in vitro model, it was shown that the serum of normoxemically resuscitated animals could prime the otherwise inert U937 monocyte like cells for the production of inflammatory cytokines. This effect was abolished when the cells were incubated with the serum drawn from animals that were resuscitated under hypoxemic conditions, highlighting a favorable systemic effect [56]. The beneficial role of this strategy in the attenuation of the systemic inflammatory response is also supported by the resulting lower serum levels of angiopoietin-2, a key player in vascular homeostasis and inflammation [57]. Systemic inflammatory response and tissue hypoperfusion following ischemia and dysregulated vascular endothelial function are the major contributors of MOF. Taken together, these results could support the hypothesis that hypoxemic reperfusion confers protection against MOF through attenuation of the inflammatory response and preservation of vascular homeostasis. Hypoxemic reperfusion has also been found to protect from lung injury and pulmonary dysfunction, which constitute a significant problem encountered after resuscitation from hemorrhagic shock [58, 59]. The favorable effects of gradual reintroduction of oxygen, include preservation of pulmonary capillary endothelial angiotensin converting enzyme activity, lower lung tissue myeloperoxidase (MPO) activity, lower lung injury histopathological score and lower MDA and intracellular adhesion molecule (ICAM) -1 and vascular cell adhesion molecule (VCAM) -1 expression levels. In another set of experiments, hypoxemic resuscitation was associated with decreased bronchoalveolar lavage (BAL) ROS levels as measured by flow cytometry as well as reduced inflammatory cytokine levels including TNF-a, IL-1b and IL-6. In addition, the nitrotyrosine score, as a marker of nitrosative stress mediated injury, was higher in the normoxemic resuscitation group of animals. Moreover, another favorable effect of hypoxemic resuscitation from hemorrhagic shock is the prevention of post-ischemic liver injury through the attenuation of nitrosative and oxidative stresses [60]. These effects were evidenced by the lower serum ROS and cytokines (TNF-a, IL-1b and IL-6) levels, the lower hepatic MDA levels and the decreased hepatic MPO and endothelial nitric oxide synthase (eNOS) and inducible nitric oxide synthase (iNOS) expression. These results, regarding the lower degree of injury of isolated organs (i.e. lung and liver) also highlight the potential beneficial role of hypoxemic reperfusion in the prevention of MOF. More recently, Luo et al. [61] compared the effects of normoxic, hyperoxic, hypoxemic and gradual resuscitation from hypoxia to hyperoxia (gradually increased oxygen administration – GIOA technique) in a rat experimental model. The authors showed that hypoxemic resuscitation resulted in worse hemodynamic profile as evidenced by the significantly lower pulse pressure as well as lower liver tissue oxygen partial pressure compared to GIOA resuscitation. The hypoxemic mode of reperfusion showed significantly lower liver injury, oxidative and inflammatory responses, only compared to hyperoxic resuscitation, while no statistically significant differences were demonstrated between GIOA and hypoxemic groups. However, the discordance between these results and previously published data [3] could be attributed to the relatively shorter follow up period in Luo’s study [61]. Longer follow up period in that study, could eventually unmask detrimental effects of the applied hyperoxemia in the context of GIOA, in contrast to normoxemia.

## Conclusions

The pivotal role of oxidative stress mediated injury after ischemia and reperfusion has been well established. Over the last decades, medical research has focused on the elucidation of the underlying pathophysiologic mechanisms in an attempt to develop strategies to attenuate this I/R injury. Hypoxemic reperfusion has been proposed as a technique aimed at the elimination of the oxidative burst that leads to the downstream cascade of free radicals and inflammation leading to multiple organ injury. This method has shown promise in various cases of I/R injury both in the experimental and clinical setting. However, further research both to clarify its underlying basic mechanisms and to assess its efficacy in the clinical setting is warranted.

## Abbreviation

ATP: adenosine triphosphate
BAL: bronchoalveolar lavage
C_a_O_2_: arterial oxygen content
CO: cardiac output
DO_2_: oxygen delivery
eNOS: endothelial nitric oxide synthase
FiO_2_: fraction of inspired oxygen
GIOA: gradually increased oxygen administration
Hb: hemoglobin
I/R: ischemia – reperfusion
ICAM: intracellular adhesion molecule
IL: interleukin
iNOS: inducible nitric oxide synthase
MAPK: mitogen activated protein kinase
MDA: malondialdehyde
MOF: multiple organ failure
MPO: myeloperoxidase
OPC: overall performance score
P_a_O_2_: partial arterial oxygen pressure
ROS: reactive oxygen species
S_a_O_2_: arterial oxygen saturation
S_ṽ_O_2_: mixed oxygen venous saturation
TNF: tumor necrosis factor
VCAM: vascular cell adhesion molecule
